# High-Efficiency Continuous Microreactors for Controlled Synthesis of Nanosized Particles of Functional Materials: Review

**DOI:** 10.3390/nano16040234

**Published:** 2026-02-11

**Authors:** Rufat Sh. Abiev

**Affiliations:** 1 Department of Optimization of Chemical and Biotechnlogical Equipment, Saint Petersburg State Institute of Technology (Technical University), 190013 Saint Petersburg, Russia; ohba@lti-gti.ru or abiev.rufat@gmail.com; 2 Konstantinov St Petersburg Institute of Nuclear Physics—Grebenshchikov Institute of Silicate Chemistry, National Research Centre “Kurchatov Institute”, 199034 Saint Petersburg, Russia

**Keywords:** microreactors, nanoparticles fabrication, inorganic materials, process intensification, micromixing, co-precipitation, oxides, phosphates, fluorides, composite materials

## Abstract

The current state and prospects of microreactor synthesis of functional materials in single- and two-phase flows with a liquid continuous phase are analyzed. Microreactors allow fine control over the size, composition, structure, and properties of synthesized particles in co-precipitation processes. The results obtained by various teams provide grounds to expect fairly extensive capabilities for controlling the processes of nucleation and particle growth in microreactors—by controlling the pH, reagent concentrations, micromixing quality, and residence time in each of the reactor zones—in the nucleation growth zones. The advantages of microreactor synthesis have been demonstrated with a high quality of micromixing in a volume of 0.2–0.5 mL, which ensures the production of nanoparticles without impurities, a stoichiometric ratio of atoms in the product, and limitation of agglomerate growth due to a short residence time (in the order of several milliseconds). The transition to an industrial scale is very easy due to the fairly high productivity of a single microreactor (up to 10 m^3^/day for suspension, up to 200–300 kg/day for solid phase). Intensive mixing in microreactors with a diameter of 2–4 mm or less, due to Taylor vortices, contributed to the use of two-phase microreactors for the synthesis of both organic and inorganic substances.

## 1. Introduction

This review is devoted to such types of reactors (and specifically microreactors) which could be used for the synthesis of nanosized particles of inorganic materials, produced by means of co-precipitation or replacement reactions, e.g., simple and complex oxides, fluorides, phosphates, etc.

This paper does not cover microreactors used for the synthesis of organic products, i.e., microreactors equipped with long microchannels, either in planar geometry or having the shape of a coiled tube.

Some types of advanced reactors with a macro scale are discussed in this paper to demonstrate their preferences in comparison with commonly used stirred tank reactors.

The novelty of this review paper consists of the summarized analysis of the diffusion and convection roles on the processes of nanoparticles synthesis, the influence of the specific energy dissipation rate on micromixing, and the comparison of commonly used as well as recently elaborated reactors (including microreactors) from a micromixing efficiency point of view.

## 2. Theoretical Background

Advantages of materials formed from nanosized particles have been widely discussed in the literature [1,2,3,4] in the last three decades, covering excellent properties due to their extremely high surface-to-volume ratio and unique quantum effects. Resulting key advantages include enhanced strength, lighter weight, improved durability, superior catalytic reactivity, and distinct optical or electrical properties compared to conventional materials.

The idea of controlling the size, composition, structure, and properties of synthesized particles in co-precipitation processes has attracted the attention of researchers in many countries [1,2,3]. In addition, high-quality homogenization of solutions at the molecular/ionic levels is also important in obtaining organometallic compounds. In [4], the kinetics of nucleation and growth of silver nanowires were studied; the Redox Crystallization model was used to describe the process.

According to the Debye–Smoluchowski theory [5], the rate of a chemical reaction in a solution is related to the concentrations of the reactants [A] and [B] according to Equation (1):

(1) $$w=k_{eff}\left[A\right]\left[B\right]$$

where *k*_eff_ is the effective rate constant, determined by the equation:

(2) $$k_{eff}=\frac{k_{react}\cdot k_{D}}{k_{react}+k_{D}}$$

In Equation (2), *k*_react_ is the true rate constant of a chemical reaction; *k*_D_ is the rate constant of diffusion mass transfer. In the literature on physical chemistry, Equation (3) is used to estimate the value of *k*_D_ [5].

(3) $$k_{D}=4\pi \left(D_{A}+D_{B}\right)\left(r_{A}+r_{B}\right)$$

where *D*_A_ and *D*_B_ are diffusion coefficients; *r*_A_ and *r*_B_ are the radii of particles A and B.

**Figure 1:**
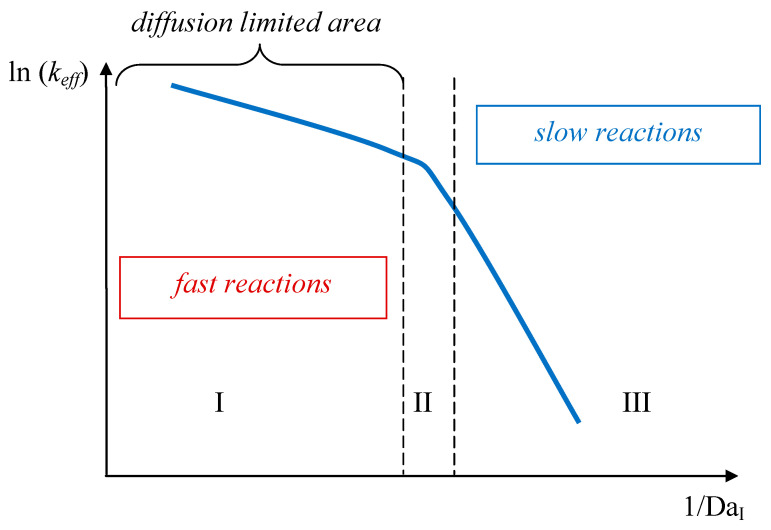
Typical dependence of the effective constant of reaction on the inverse Damköhler number: I—diffusion-limited area; II—transient area; III—reaction kinetic-limited area.

Figure 1 shows a typical dependence of the effective constant of reaction on the inverse Damköhler number, calculated as follows:

Da_I_ = (reaction rate)/(convection mass transfer rate) (4)

For fast reactions, which include neutralization, substitution, and a number of others that occur in co-precipitation processes, the following relationship is valid:

(5) $$k_{D}<<k_{react},$$

For instance, the effective rate of the process is fully determined by the diffusion, or more precisely, by the convective-diffusion factor.

Equation (2) is, in general, applicable both to the molecular (or ionic) level of particles and to the level of nanosized particles capable of diffusion transfer. For larger particles (e.g., submicron agglomerates or aggregates), along with the diffusion transfer, convective transfer should be considered, the intensity of which is determined by the quality of mixing at the micro, meso, and macro levels.

It is known that at high Peclet numbers (Pe = *UL*/*D*), convection is the predominant mechanism of substance transfer compared to diffusion. Here, U is the characteristic velocity of liquid movement, m/s; L is the characteristic size of the apparatus, m; and D is the coefficient of diffusivity, m^2^/s.

**Figure 2:**
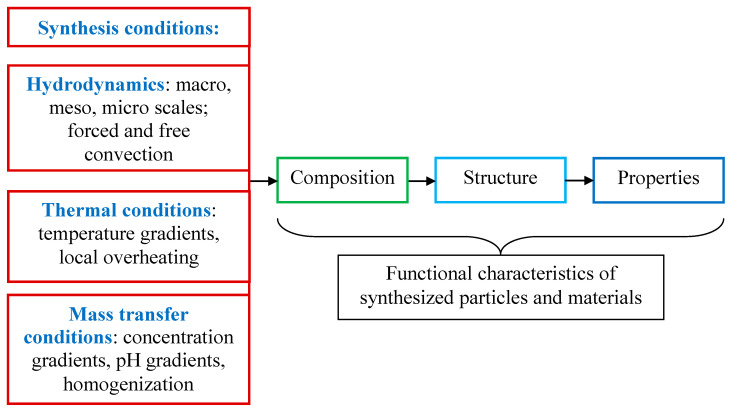
Schematic of the synthesis conditions’ influence on the composition, structure, and properties of functional materials for liquid-phase synthesis stages: sol–gel, co-precipitation, hydrothermal, and solvothermal methods.

Usually, in publications, during analysis of the synthesis conditions in co-precipitation processes, the convection-diffusion factor is given very little attention. Meanwhile, as follows from Equations (1)–(5), for synthesis in solutions, convection and diffusion have a significant effect on heat and mass transfer processes, on the uniformity of reagent distribution in the solution volume, on local pH values, and also on local supersaturations. All these processes dramatically affect the synthesis conditions, determining not only the kinetics of nucleation and particle growth but also the composition, morphology, and properties of particles and materials based on them. The scheme of factors influencing the characteristics of functional materials obtained in solutions is shown in Figure 2.

Evidently, after synthesis, the separation of the particles from the mother liquor is the next important step in the technology of nanosized and submicron particle production. Long residence times of particles in the growth solution lead to aggregation and further agglomeration of particles, resulting in a decrease in their preferences relative to common synthesis methods. This is why the particle separation step should be performed in a safe and quick mode in order to suppress the uncontrolled growth of particles. It should be noted, however, that in some cases (like the production of ceramics), the secondary growth of particles is needed for further stages of production. Usually, three methods of particle separation from the mother liquor are employed for this aim at a lab scale, such as the following: (i) filtration, (ii) decantation with liquid addition and removal (washing), or (iii) centrifugal separation. All three methods can be scaled up to the industrial level. A detailed discussion on particle separation is out of the scope of this paper and can be found in the literature [3,4,5].

Ostwald ripening involves a change in an inhomogeneous structure over time, in that small crystals or sol particles first dissolve and then redeposit onto larger crystals or sol particles; particle growth processes like aggregation and Ostwald ripening need time. Therefore, nucleation dominates in microreactors with very short residence times.

One of the appealing advantages of microreactors is the much higher surface-area-to-volume ratio (relative to common size reactors). This feature has a direct influence on particle formation in nanoparticle synthesis; due to the much shorter diffusive path, the ions of reagents are able to form nuclei, which then rapidly grow to the desired 10–100 nm size.

For organic compounds, the production of which is associated with the formation of explosive products, the use of microreactors has another advantage—the channel diameter can be taken to be less than the critical one for a given group of substances. It should be noted that several tens of thousands of parallel microchannels can be used in an industrial microreactor, i.e., the name here is due to the transverse size of the channels (about 2–4 mm or less), and the productivity is comparable to that of standard types of chemical equipment.

The aim of this work is to demonstrate the advantages and promising possibilities of inorganic functional materials synthesis in microreactors specially developed for these purposes.

In this paper, several classes of microreactors for the synthesis of nanosized particles of inorganic materials are discussed as follows: (i) for synthesis in single-phase liquid media and (ii) for synthesis in two-phase liquid–liquid systems.

## 3. Concept of Co-Precipitation at High-Quality Micromixing (Flash Co-Precipitation Method)

The idea of using intensive micromixing, especially in microreactors that have various geometries and principles of flow mixing, is the focus of several tens of papers within the last two decades [1,2,3,4].

In [6], some of the new methods of nanoparticles synthesis are discussed, such as (i) a common stirred tank reactor with a Rushton turbine, (ii) a two impinging-jets mixing device with an angle of 180° between the impinging jets, (iii) two impinging-jets mixing device with an angle of about 30° between the impinging jets, (iv) a stirred tank reactor with a sliding disc located just near the bottom (with a gap between 0.5 mm and 3.0 mm) and reactant supply under the disc, (v) and a vortex reactor equipped with a propeller in the bottom zone and two pipes for a liquid reactant supply in the upper zone of the reactor.

**Figure 3:**
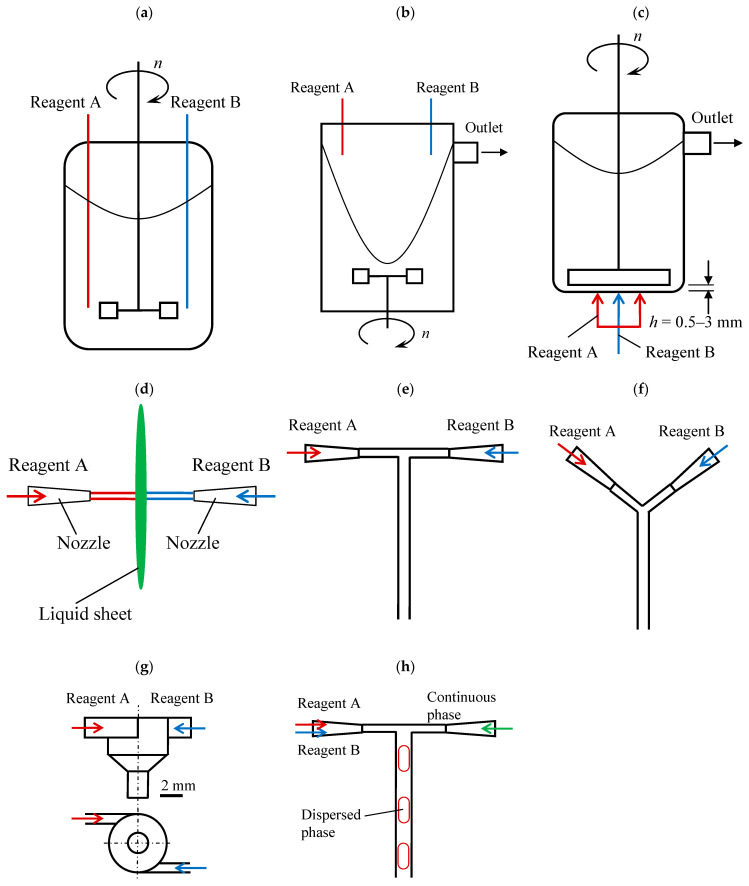
Typical reactors used to fabricate nanosized particles: (**a**)—CSTR; (**b**)—two-jets vortex reactor; (**c**)—sliding surface mixing device; (**d**)—impinging-jets mixing devices; (**e**)—T-mixer; (**f**)—Y-mixer; (**g**)—Hartridge–Roughton reactor (cyclone-type mixer); (**h**)—two-phase segmented flow microreactors.

A comparison of these and some other types of microreactors most frequently used for the synthesis of nanosized particles is presented in Table 1, and their schematic diagrams are shown in Figure 3.

The Continuous Stirred Tank Reactor, also called the “Standard configuration of the precipitation reactor” (Figure 3a), is characterized by large-scale primary turbulence generated by the impeller (e.g., Rushton turbine or pitched blade turbine, PBT). The cascade-like breakup of turbulent vortices has a negative influence on particle nucleation, as discussed in the next paragraph. Possibly the only effective improvement for this reactor is the use of feeding tubes located just near the impeller.

The Continuous Stirred Tank Reactor, due to a very low quality of agitation, has zones with supersaturation levels where nucleation preferentially occurs, whereas growth may continue in zones of moderate supersaturation [6]. Moreover, nucleation can continue in parallel with growth, and if agglomeration occurs, this will lead to a wider particle size distribution. Low quality of agitation in CSTRs is caused by an extremely non-uniform distribution of specific energy dissipation rates. Moreover, the location of feeding points plays a significant role in the particle formation processes in the CSTR. For example, locating the feed points near the surface generally gives birth to high amounts of agglomerates; this effect is obviously caused by the ultimately low quality of micromixing on the liquid surface [7]. On the contrary, the location of the feeding pipes within the radial stream of the stirrer leads to very few agglomerates [6].

These drawbacks of CSTR motivated researchers to create new types of reactors that are better suitable for the nucleation and growth of nanosized particles so as to provide narrow size distributions.

The two-jet vortex reactor (Figure 3b) differs from the CSTR in the liquid supply manner: two jets of reacting liquid are fed to the liquid surface from the upper side of the reactor, whereas the impeller (usually a Rushton turbine) is located in the bottom part of the reactor. The rotation of the impeller in a tank without baffles leads to the generation of an active central vortex having a depth comparable with the reactor height (also called a “free vortex”). The reacting fluids are injected into the free vortex, and nucleation takes place totally in this vortex; this results in the absence of incrustation on solid surfaces [7]. Even though the rotation of liquid caused by the stirrer seems to improve mixing in general, the level of energy dissipation rate in the upper part of the liquid is several orders of magnitude lower compared to that immediately near the stirrer [7]. Therefore, the only advantage of such a device is apparently the almost direct contact of mixed solutions, and the long residence time leads to ripening, i.e., the formation of quite large aggregates in the reactor. In some technologies, however, the production of aggregates is preferred (for instance, in the production of ceramics).

The sliding surface mixing device (Figure 3c) appears to be an advanced solution compared to the two-jet vortex reactor because solutions are fed directly into the area of most intensive mixing. i.e., just in the small gap (0.5–3 mm) between the rotating disk (stirrer) and the bottom of the reactor. The rotating disk has a large diameter (0.8…0.9 of the tank diameter) and rotates with a high speed (20…50 s^−1^, i.e., 1200…3000 rpm). The confined mixed zone situated under the disc allows for rapid and intensive mixing in the small gap under the disc; the upper zone is moderately mixed by the disc, and here the growth of particles takes place. One of the solutions is fed to the center of the device, and the second one is supplied to the periphery of the stirrer; due to centrifugal effects and high shear stresses in the small gap, solutions are better mixed. The other parts of the reactor have, however, much lower levels of energy dissipation, and heavy particles (with negative buoyancy) can concentrate in the bottom part of the reactor, leading to clogging.

In impinging-jets mixing reactors (Figure 3d), two free liquid jets, usually with a diameter of 1 to 3 mm and a velocity in the range from 3 to 10 m/s, collide (impinge) in the ambient gas, forming a liquid sheet with a thickness of about 100 μm. The primary mixing and synthesis take place in a small volume located in the area of jet impingement (having a diameter slightly larger than the diameter of the jets); afterwards, the mixed solution moves radially, and at the border of the liquid sheet, due to velocity deceleration, a liquid rim is formed around the sheet. The authors of work [6] claim that in impinging-jets mixing devices (Figure 3d), “obstruction problem cannot occur”. Actually, this point has the reverse side, as it was shown in studies [8,9] (see also references within); the mixing quality in the liquid sheet varies for different zones, and at high velocities of the jets, the behavior of the liquid sheet in “free volume” leads to the formation of filaments having much worse quality of micromixing.

In T-mixers (Figure 3e), Y-mixers (Figure 3f), and similar devices (like X-mixers, co-flow mixers, etc.), they are actually part of microreactors where the first contact of solutions takes place, and then they mix downstream. There are a variety of microreactors combining T- and Y-mixers with a downstream microchannel having complex geometries, and quite high mixing efficiencies are achieved in them. The T- and Y-mixers can be considered as a kind of microreactor with submerged jets, but they have a small diameter in the mixing chamber, equal to the diameter of the inlet pipes. It is worth mentioning that for flash processes having very short reaction times, the main part of co-precipitation can take place in the small volume of these mixers, where two inlet flows (submerged jets) collide with one another. In other words, for very fast (almost instant) reactions (co-precipitation, substitution reactions), the quality of mixing in downstream microchannels does not play any significant role if the residence time in T- and Y-mixers is larger than the reaction time.

For other cases, like the formation of nanoparticles in droplets of two-phase flows (see Figure 3h), having a larger reaction time, the downstream microchannels with complex geometries are important. Actually, the microreactor with pulsating flow (MRPF), discussed below, also belongs to this group of microreactors.

T-mixers (Figure 3e) and Y-mixers (Figure 3f) are widely used in experiments due to their extremely simple design. Still, in fact, the level of mixing achieved in these devices is quite low, as was shown in [10]: the segregation index determined by use of the iodide-iodate method was slightly better compared to CSTR; however, it was much worse than that obtained in microreactors with swirling flows described in the present paper below.

In Hartridge–Roughton reactors (the other name is “Cyclone type mixers”, Figure 3g), there are two tangential inlet pipes attached to the main body of the microreactor; a vortex is formed in such a microreactor due to rotations caused by two inlet flows. This kind of vortex mixer has, therefore, excellent mixing characteristics [11]. The typical segregation index was found to be lower than 0.025 for inlet flow rates of 5 L/h, and mixing times were less than 5 ms [11].

The Hartridge–Roughton reactor is, in our opinion, one of the most advanced microreactors from a mixing efficacy point of view because of the intensive swirling concentrated in a quite small volume: the typical size of the inlet pipes is ~1 mm, and the outlet pipe has a diameter of 2 mm. In some works [11], even smaller sizes of Cyclone-type mixers (about 100 μm) have been used. Such small sizes result in very limited throughput (<15 L/h). Moreover, the first contact of solutions takes place in the upper volume of the reactor, having the lowest energy dissipation rate. This, in turn, results in a lesser quality of micromixing compared to the microreactors with intensively swirled flows (MRISF) described in this paper; see also [12] for details.

Two-phase segmented flow microreactors (Figure 3h) are a promising tool for interphase synthesis of organic products, but synthesis of inorganic particles in two-phase segmented flow attracts the attention of researchers as well (see references in the last row of Table 1). The main preference of such reactors is the formation of segmented flow containing elongated droplets of dispersed phase (often called “plugs”) moving between droplets of continuous phase (called “slugs”). Each plug is separated from the others so that each droplet behaves as a moving nanoliter reactor; the same applies to the slugs. Under some conditions (capillary number should be smaller than 0.7), Taylor vortices appear inside both plugs and slugs, resulting in much better mixing compared to one-phase laminar flow. Mixing takes place due to Taylor vortices caused by the velocity profile in each phase and due to the limited length of droplets. Several materials have been successfully produced in such conditions. The drawbacks of such a method are limited mixing intensity and a typical residence time that varies from ~1 min to ~10 min, which could be too much for some fast reactions.

Ultrasound was used in some works to improve mixing. Kale et al. [13] applied a microreactor with ultrasound sonication for the continuous production of TiO_2_ nanoparticles. It has been revealed that the reagent concentration, flow rate, and geometry of the microreactor play significant roles in the production of nanoparticles. Ultrasound-assisted continuous processing in a microreactor was studied in [14], significantly affecting crystallization and chemical synthesis, resulting in improved efficiency, yield, and product quality.

Moreover, TiO_2_ nanoparticles have been effectively produced in a sonicated ultrasound-assisted continuous mini reactor by Wagner et al. [15] in a water-in-oil emulsion; precise control over particle size and distribution was achieved.

Finally, some attempts to model the physics of ultrasound-assisted microreactors have been undertaken by several researcher groups [16,17].

**Table 1:** State-of-the-art for reactors used for the synthesis of nanosized particles by (co-)precipitation from reactant solutions.

No.	Type of Reactor	Features (Pros and Cons)	References, Estimated * Range of Specific Energy Dissipation Rate, W/kg
1	Continuous Stirred Tank Reactor (CSTR), Figure 3a	**Pros:** Easy to order from Bulk Chemical Engineering, with a wide range of volumes and sizes **Cons:** Micromixing quality is limited by the level of the specific energy dissipation rate uniformity Increased risk of secondary nucleation, wider particle size distribution	[6] ε_av_ = 0.3…3 W/kg, ε_max_ = 34ε_av_ [7]
2	Two-jet vortex reactor, Figure 3b	**Pros:** Better mixing compared to CSTR, but the most intensive mixing takes place in the zone near the bottom, whereas the solutions are supplied through the liquid surface **Cons:** Reactions can take place at the liquid surface	[6] ε_av_ = 0.5…5 W/kg, ε_max_ = 10ε_av_
3	Sliding surface mixing device, Figure 3c	**Pros:** Nice mixing near the bottom, and the flows are injected into this area **Cons:** The zone over the stirrer has a lower level of mixing, but if the reactions occur in the lower zone, it is not an issue	[18,19] ε_av_ = 0.3…3 W/kg, ε_max_ = 50ε_av_
4	Impinging-jets mixing devices, Figure 3d	**Pros:** Concentrated energy dissipation in the impingement micro-volume **Cons:** Limitations of flow rates: the liquid sheet disintegrates at high velocities; flow rates of jets should be equal	[20] ε_av_ = 10^2^…10^3^ W/kg [9] ε_max_ = 10ε_av_
5	T-mixers, Figure 3e	**Pros:** Easy design of the reactor, residence time could be adjusted by the length of the reactor, and the intensity of mixing is directly affected by the liquid’s velocity. Moderate mixing efficiency is defined mainly by the turbulence generated in the inlet pipes’ junction and in the main pipe of the mixer **Cons:** For a laminar regime of flow, secondary vortices improve the quality of mixing, but for precipitation processes, this intensity may not be enough	[1,21,22,23,24,25] ε_av_ = 8…50 W/kg
6	Y-mixers, Figure 3f	**Pros and cons:** Similarly to T-mixers	[26] ε_av_ = 8…50 W/kg
7	Hartridge–Roughton reactor, Cyclone-type mixers, Figure 3g	**Pros:** Very nice quality of mixing due to intensive vortices Mixing takes place in a small volume, leading to better micromixing quality compared to stirred reactors **Cons:** Limited throughput (<15 L/h) due to extremely small sizes of the reactor. First contact of reacting solutions takes place in a volume with a relatively low-energy dissipation rate	[13,27,28,29] ε_av_ = 5…20 W/kg
8	Two-phase Segmented Flow microreactors, Figure 3h	**Pros:** Synthesis takes place in nanoliter droplets of dispersed or continuous phase (nano-reactors). Nucleation and growth occur inside droplets. Taylor vortices intensify mixing quality. Adjustable residence time (usually from ~1 min to ~10–30 min) **Cons:** Risk of clogging in case of formation of large particles (aggregates and agglomerates). The quality of mixing is limited by the velocity of the phases; Taylor flow should be kept up.	[30,31,32,33] ε_av_ = 0.003…32 W/kg

* Data have been either directly collected from the literature or assessed by the author using information on the angular velocity of the stirrer *n*, velocity of flows *U*, and from the other characteristics of the device (ε_av_—average value for the total reactor volume; ε_max_—maximal value for the zone with the most active mixing). It should be noted that the specific energy dissipation rate strongly depends on *n*^2^ or *U*^2^ for laminar flow and on *n*^3^ or *U*^3^ for turbulent flow.

**Figure 4:**
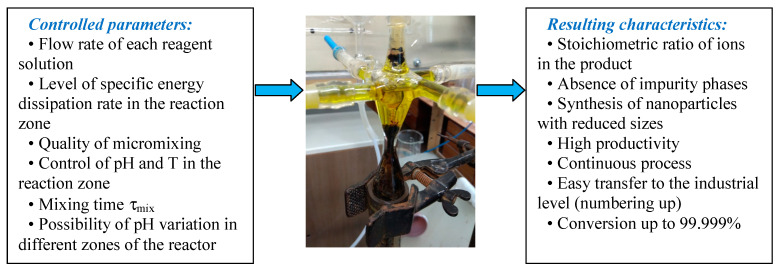
Concept of controlled synthesis of nanosized particles in microreactors: left—input parameters; right—output parameters; middle—microreactor (as an example, MRISF-1 is presented here).

The concept of controlled synthesis of nanosized particles in microreactors is shown in Figure 4: inlet parameters are shown in the left part. It is worth noting that the small-size reactors (microreactors), like the one shown in Figure 4 or similar ones discussed further, have an excellent quality of micromixing: a segregation index down to 0.002 and even less could be achieved at high liquid flow rates, i.e., up to 250 times better compared to the mixing in a stirred lab-scale reactor. Interestingly, the microreactors discussed in this paper are characterized by quite high performance, up to 10 m^3^/day (420 L/h) for suspension; for a concentration of 20–30 g/L, this corresponds to the productivity of 200–300 kg/day for nanosized particles. Energy consumption for two flow supplies is not larger than 20–30 W.

**Figure 5:**
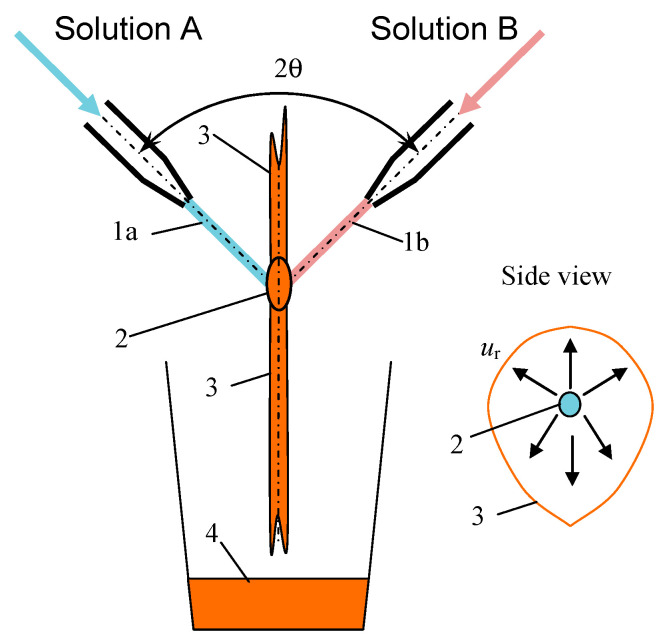
Schematic diagram of a microreactor with free impinging jets: 1—jets of reagent solutions (1a) and precipitant (1b); 2—jet collision zone; 3—liquid sheet; 4—suspension with particles; *u_r_*—radial velocity component.

**Figure 6:**
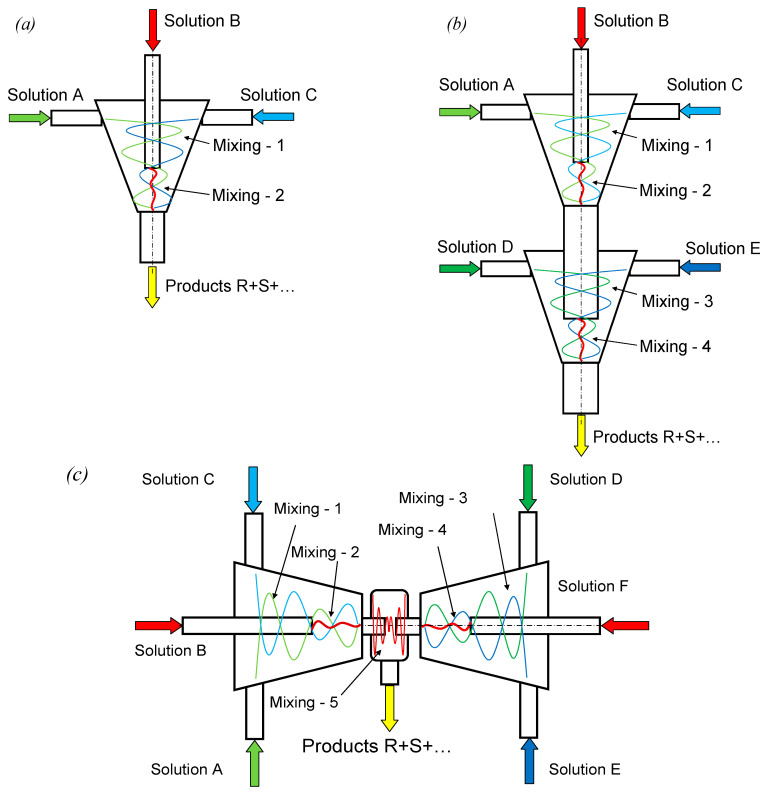
Schematics of microreactors with intensively swirling flows (MRISF): (**a**)—single-stage MRISF-1; (**b**)—two-stage MRISF-2; (**c**)—with counter-current swirling flows, MRISF-CC-1. A–F—solutions of reagents; R + S + …—suspension with products of reactions; Mixing-1–Mixing-5—mixing zones.

**Figure 7:**
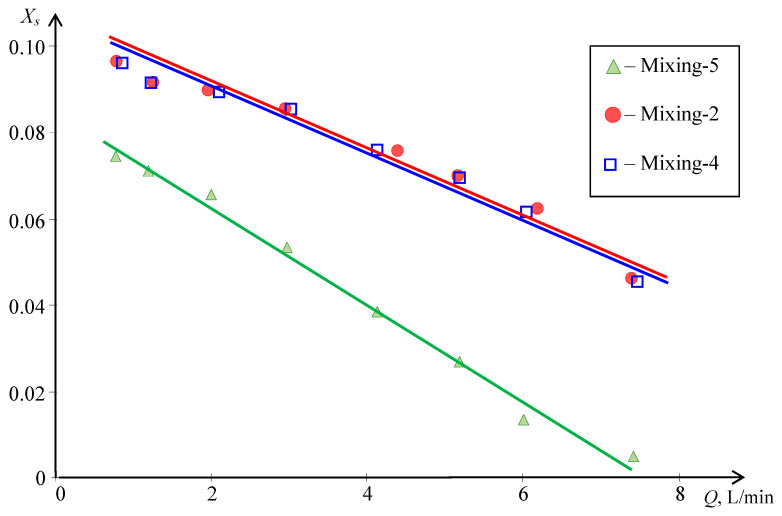
Dependencies of the segregation index *X_s_* on the total flow rate *Q* for three chambers of the microreactor with counter-swirling flows, MRISF-CC-1: chambers “Mixing-2”, “Mixing-4”, and “Mixing-5”.

Among the microreactors for nanoparticles synthesis in single-phase liquid media, which is a type of co-precipitation with intensive mixing, the following types can be distinguished: (1) microreactors with free impinging jets (MRFIJ), including those with pulsating jets [8,9] (Figure 5); (2) microreactors with intensively swirling flows (single- and two-stage, with counter-swirling flows) [10,12] (Figure 6); (3) a pulsating flow-type microreactor (which can also be effectively used for synthesis in two-phase flows; Figure 7). In these microreactors, the local value of the specific energy dissipation rate ε in the reaction zone is up to 45–48 kW/kg, which is 3–4 orders of magnitude higher than in stirred reactors (for these apparatuses ε rarely exceeds 1–3 W/kg) [10,12,34].

In microreactors with free impinging jets (MRFIJ), shown in Figure 5, two liquid jets, 1a and 1b, collide in the atmosphere, forming a liquid sheet (3), in which intensive micromixing takes place, especially in the small zone 2 [8,9,35].

Nanosized particles of bismuth and gadolinium orthoferrites, as well as LaPO_4_, Y_3_Al_5_O_12_, and CoFe_2_O_4_, as well as magnesium hydrosilicate, have been synthesized in MRFIJ. The size of GdFeO_3_ nanoparticles necessary for magnetic resonance tomography should not exceed 30 nm; in works [8,9], nanoparticles with a size of 25 nm were produced. The present case serves as a sample of flash synthesis conducted in microreactors, thereby yielding nanoparticles with a smaller size in comparison to other synthesis techniques.

It should be noted that MRFIJ has limitations similar to those mentioned above for impinging-jets mixing devices in general (see Table 1): too high a velocity of jets results in excess kinetic energy of jets, and this, in turn, leads to the precocious rupture of liquid sheet 3 (see Figure 5). Consequently, the size of the liquid sheet decreases too much for the jets with a velocity higher than 5–8 m/s (a value typical for water solutions; we have checked the behavior of the liquid sheet for jet velocities up to 40 m/s) and the liquid sheet disintegrates quickly onto filaments, splashes, and droplets, having very wide volumes and an intensity of mixing. Thus, the performance of the impinging-jets reactors (including MRFIJ) is limited by the diameter of jets (usually not larger than 2.0–2.5 mm) and by the velocity of jets (<5–8 m/s).

Microreactors with intensively swirling flows (various versions of MRISF) have been elaborated recently (see Figure 6). Their specific preferences in process intensification are founded on the acceleration of swirling in the neck of the microreactor as well as on the concentration of both kinetic energies of axial and tangential movement in a very small volume (from 0.2 to 0.5 mL) with an extremely high level of micromixing. It has been demonstrated that even in the single-stage microreactor MRISF-1 or two-stage microreactor MRISF-2, the quality of micromixing strongly depends on the specific energy dissipation rate ε (unlike micromixing in stirred tank reactors). The index of segregation falls from *X_s_* = 0.01 at low ε values to *X_s_* = 0.002 at higher ε values (in STR, *X_s_* = 0.5 and is independent of the specific energy dissipation rate in the range of rotary speed from 200 to 1000 rpm).

In a recent paper [34], a detailed comparison of MRISFs with microreactors with submerged impinging jets (MRSIJ) having the same geometry but with only a difference in the method of liquid supply (in the latter case, the tangential inlet pipes have been replaced by radial ones) has been studied both numerically and experimentally. Interestingly, at the same flow rates, MRSIJ has a larger total energy consumption compared to MRISF, but the local specific energy dissipation rate in the neck of MRISF is much higher, and consequently, the quality of micromixing is better as well. Hence, the intensively swirling flows have shown more attractive properties for applications in chemical technology.

A microreactor with counter-swirling flows, MRISF-CC-1 (see Figure 6c), contains five chambers with intensive micromixing, and three of them (Mixing-2, Mixing-4, and Mixing-5 in Figure 6c) demonstrate an extremely high level of micromixing [35]. In the chamber “Mixing-5” with impingement of the counter-current swirled flows, even better micromixing quality (lower *X_s_* values) compared to the chambers, such as “Mixing-2” and “Mixing-4”, has been observed (see Figure 7).

Microreactors with intensively swirling flows have surely a list of limitations: (i) they are suitable for very fast reactions (with a shorter reaction time than residence time, i.e., <10 ms); (ii) due to a very short residence time, it is better to preheat solutions of reagent to the reaction temperature; (iii) the ratio of flow rates supplied through various inlet pipes is limited approximately by 3–5, however, this issue could be circumvented by a distributed liquid supply through two to six or even more inlet pipes (this possibility has already been checked in the author’s lab).

**Figure 8:**
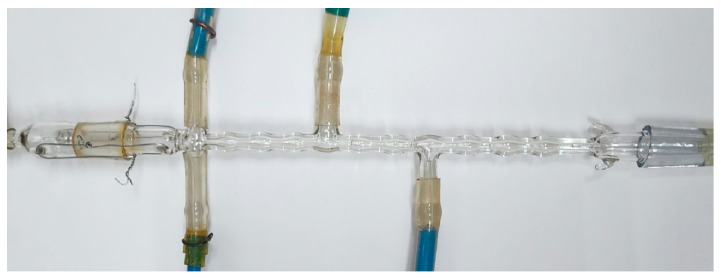
Photograph of the microreactor with pulsating flows (MRPF).

Another principle of micromixing intensification is realized in the microreactor with pulsating flows (MRPF), which is shown in Figure 8. Two or more flows can be fed through the inlet pipes shown on the left side of Figure 8, with the outlet through the pipe at the right end of the device. Pulsations in this microreactor are generated in the flowing liquids due to repeating expansions and contractions of the cross-section area, leading to periodic variations in velocity, pressure, and shear stresses. Studies of micromixing quality in MRPF have been carried out recently (the paper is under preparation) and have shown a moderate quality of micromixing compared to microreactors with intensive swirled flows. MRPF, however, has excellent features for mass transfer and micromixing intensification in two-phase flows, which are an attractive tool for several types of fast and moderately slow chemical reactions.

Limitations of the MRPF reactor, in general, coincide with the disadvantages of two-phase segmented flow microreactors (see Figure 3h): Taylor flow should be created in this microreactor, and the mixing is defined by Taylor vortices, whose intensity is limited by the velocity of the phases. Moreover, some risks of clogging in case of formation of large particles still exist, but due to the larger diameter of the MRPF microreactor (we have used such a device with the smallest diameter of 2 mm), such risks are minimal. Pulsations created in this type of microreactor decrease the probability of clogging as well.

The specific energy dissipation rate ε is one of the key characteristics of chemical reactors that determine the quality of mixing at all levels—macro, meso, and micro—as well as directly affecting the processes of heat and mass transfer. However, along with the specific energy dissipation rate, the characteristics of mixing and mass transfer are influenced by the design of the apparatus [36], as well as the place of reagent injection and the area of their primary contact [37,38]. In [36], the quality of micromixing in eight types of microreactors (two types of T-mixers, Tangential IMTEK mixer, Caterpillar micromixer (IMM), Standard slit interdigital micromixer (IMM), Triangular interdigital micromixer (Mikroglas), K-M mixers, and Starlam (IMM)) was analyzed; they all have excellent quality of micromixing. However, the spread of micromixing time at the same values of ε, for different types of microreactors, was up to 1–2 decimal orders in [36]. The microreactors shown in Figure 6, as well as their modifications, were designed and executed taking into account all the above factors, which allowed us to achieve favorable synthesis conditions.

High quality of micromixing is an important (and not yet sufficiently evaluated) prerequisite for the formation of nanoscale particles with a given purity and size, and potentially with a given structure [37,38].

Micromixing is the most subtle stage of mixing, close to the molecular level. It consists of visco-convective deformation of liquid elements, which accelerates the disintegration of liquid aggregates up to the diffusion scale [36,37,38]. It is at this level that the selectivity of reactions is determined. This mechanism includes the involvement (English: “engulfment”) and deformation of vortices of the Kolmogorov scale λ_k_ and is a limiting process in reducing local concentration gradients. Another quantitative characteristic of the process is the micromixing time associated with the specific energy dissipation rate ε: the higher ε, the better the micromixing and the higher the selectivity of fast-flowing reactions. According to the results of data processing for eight types of microreactors, the average micromixing time, *t*_m_, is related to the specific rate of energy dissipation by the ratio [36]:

(6) $$t_{m}=0.15\;\varepsilon^{-0.45}$$

Micromixing time, taking into account the diffusion factor (Schmidt number), is defined as [36]:

(7) $$\;t_{m}={\left(\frac{\nu }{\varepsilon }\right)}^{\frac{1}{2}}\mathrm{arcsinh}(0.1\;\mathrm{Sc})$$

where ν is the kinematic viscosity of the liquid, m^2^/s; Sc = ν/D is the Schmidt number.

It follows from formulas (5) and (6) that *t_m_* ~ (ε^−0.45^ … ε^−0.5^), i.e., an increase in the specific rate of energy dissipation by a factor of 10 leads to a decrease in the micromixing time by 2.82…3.16 times. It is easy to find that with an increase of ε from 3 W/kg (for a device with a stirrer with intensive stirring) to 48 kW/kg (for microreactors with intensively swirled flows), the micromixing time decreases by about 125 times and amounts to ≈3.5 ms! Thus, the microreactors ensure mixing within a few milliseconds. It is important to note that the residence time in the neck of microreactors with intensively swirling flows is approximately 7–10 ms, i.e., during this time, micromixing, nucleation, and some particle growth occur, after which the particles are removed from the apparatus. Then, measures must be taken to prevent their excessive growth.

## 4. Practical Applications of Microreactors for Nanoparticle Synthesis

### Examples of Nanoparticle Synthesis in Microreactors with Intensive Micromixing

More than 20 types of inorganic materials have been synthesized in microreactors with intensive micromixing—in MRFIJs and MRISFs. Some examples of such materials and references are shown in Table 2. In all cases, the particles with sizes under 100 nm (typically 20–40 nm) have been produced; aggregates of particles formed in MRFIJs and MRISFs are much smaller compared to those produced in the usual way (in the stirred reactors).

## 5. Synthesis of Single and Complex Oxides

**Figure 9:**
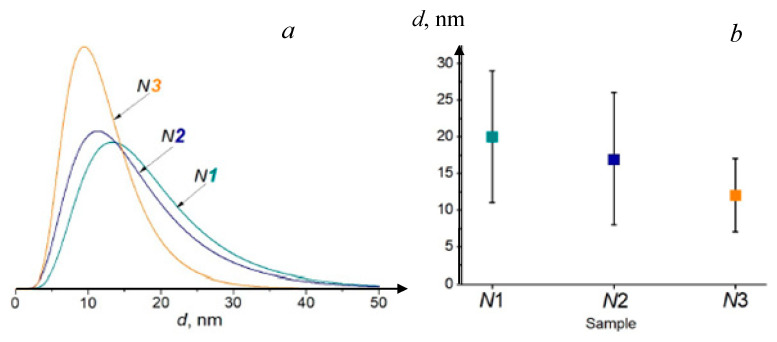
Synthesis of BiFeO_3_ by use of three microreactors (*N*1—microreactor with submerged impinging jets MRSIJ; *N*2—microreactor with free impinging jets MRFIJ; *N*3—microreactor with intensively swirled flows MRISF-1): (**a**)—average sizes of bismuth orthoferrite crystallites in samples after heat treatment; (**b**)—size distribution of crystallites according to the 012 reflection in samples *N*1, *N*2, and *N*3; *d_avN1_* = 19 nm, *d_avN2_* = 17 nm, and *d_avN3_* = 12 nm. Standard deviation σ*_N1_* = σ*_N2_* = 9 nm and σ*_N3_* = 5 nm.

**Figure 10:**
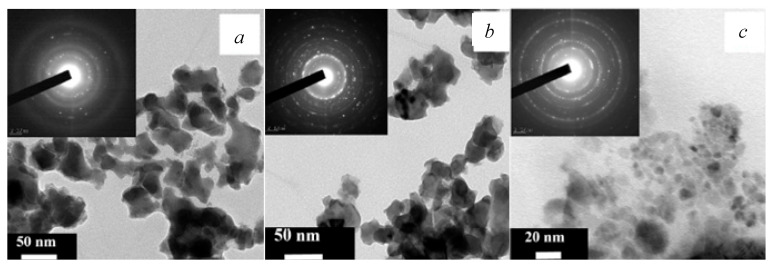
TEM photographs of BiFeO_3_ nanoparticles after heat treatment: (**a**)—microreactor with submerged impinging jets, MRSIJ (*N*1); (**b**)—microreactor with free impinging jets, MRFIJ (*N*2); (**c**)—microreactor with intensively swirled flows, MRISF-1 (*N*3).

A comparison of BiFeO_3_ properties produced by the use of three microreactors is presented in Figure 9 [39]: *N*1—a microreactor with submerged impinging jets MRSIJ; *N*2—a microreactor with free impinging jets MRFIJ; *N*3—a microreactor with intensively swirled flows MRISF-1. TEM photographs of BiFeO_3_ nanoparticles after heat treatment for all three methods are shown in Figure 10. As can be seen in Figure 9 [39], the mean diameter of the particles for these three methods is *d_avN1_* = 19 nm, *d_avN2_* = 17 nm, and *d_avN3_* = 12 nm, and the standard deviation is σ*_N1_* = σ*_N2_* = 9 nm and σ*_N3_* = 5 nm. This example shows that the microreactor with free impinging jets demonstrates better quality compared to the MRSIJ; however, the microreactor with intensive swirled flows, MRISF-1, has an even smaller mean diameter of particles (*d_avN3_* = 12 nm) and a lesser standard deviation (σ*_N3_* = 5 nm). These differences can be explained by the effect of micromixing quality: (i) in a microreactor with submerged impinging jets, the energy of jets is dissipated in quite a large volume of surrounding liquid, and the specific energy dissipation rate is relatively low; (ii) in a microreactor with free impinging jets flow, the specific energy dissipation rate is larger compared to the MRSIJ; moreover, there is a small volume of colliding jets, but the solution with reaction products move radially within liquid sheet, where some growth of particles evidently happens; (iii) in a microreactor with intensive swirled flows, the specific energy dissipation rate is the highest among these three microreactors, and the residence time in a small mixing volume is very short; both factors result in smaller average size and narrower particle size distribution for MRISF-1.

**Figure 11:**
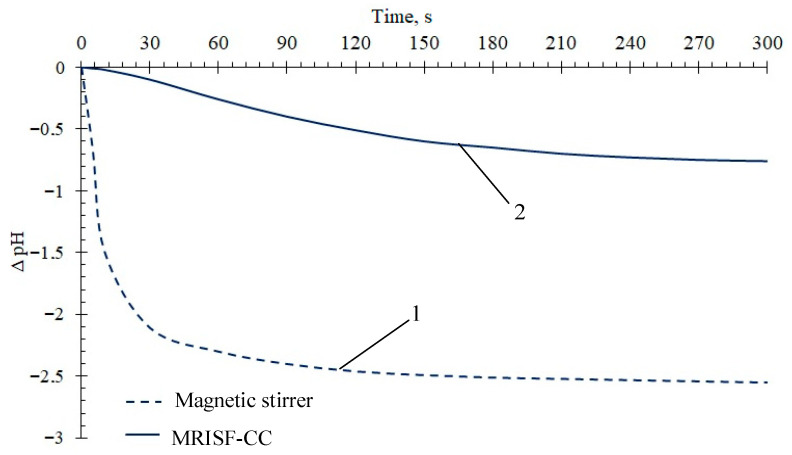
Kinetics of pH for ZrO_2_-Y_2_O_3_ xerogels in water, synthesized by means of co-precipitation in the stirred reactor (with magnetic stirrer) (line 1) and by means of fast co-precipitation in the MRISF-CC-1 (line 2).

Synthesis in the microreactor MRISF-CC-1 of ZrO_2_, stabilized with Y_2_O_3_, was studied in [43]; the size of agglomerates after synthesis and drying was just ≈380 nm, i.e., 1.6 times smaller compared to agglomerates produced by the usual method of co-precipitation in a stirred reactor. Interestingly, the xerogels produced by use of co-precipitation in the stirred reactor demonstrated a predominance of Lewis acid centers, whereas the xerogels produced by use of fast co-precipitation in the MRISF-CC-1 possess Brønsted acid centers (Figure 11), i.e., the excess of OH-ions on the surface of particles is observed in the latter case.

**Figure 12:**
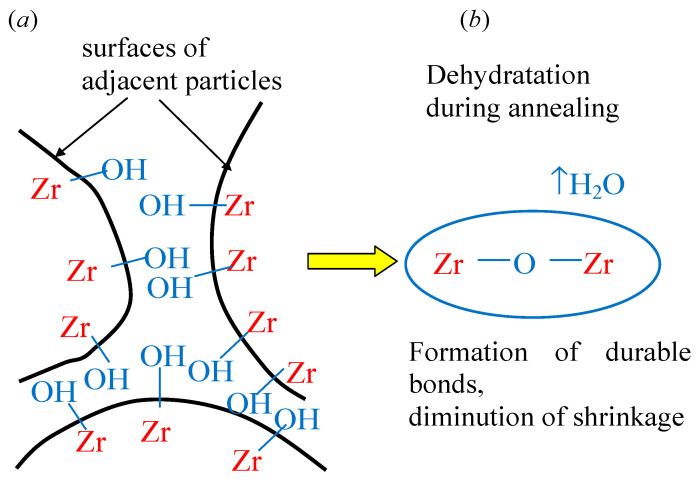
Super-hydroxylation of the particles’ surface during co-precipitation in microreactor MRISF-CC (**a**), leading to the formation of durable Zr-O-Zr bonds between particles during annealing, leading to the diminution of materials shrinkage (**b**). The yellow arrow shows transitions in material after annealing.

Apparently, after subsequent thermal treatments, these OH-groups will form Me-O-Me bonds, leading to an increase in the durability of ceramics, as shown in Figure 12 (here, “Me” designates metal ions).

## 6. Synthesis of Calcium and Strontium Fluorides

**Figure 13:**
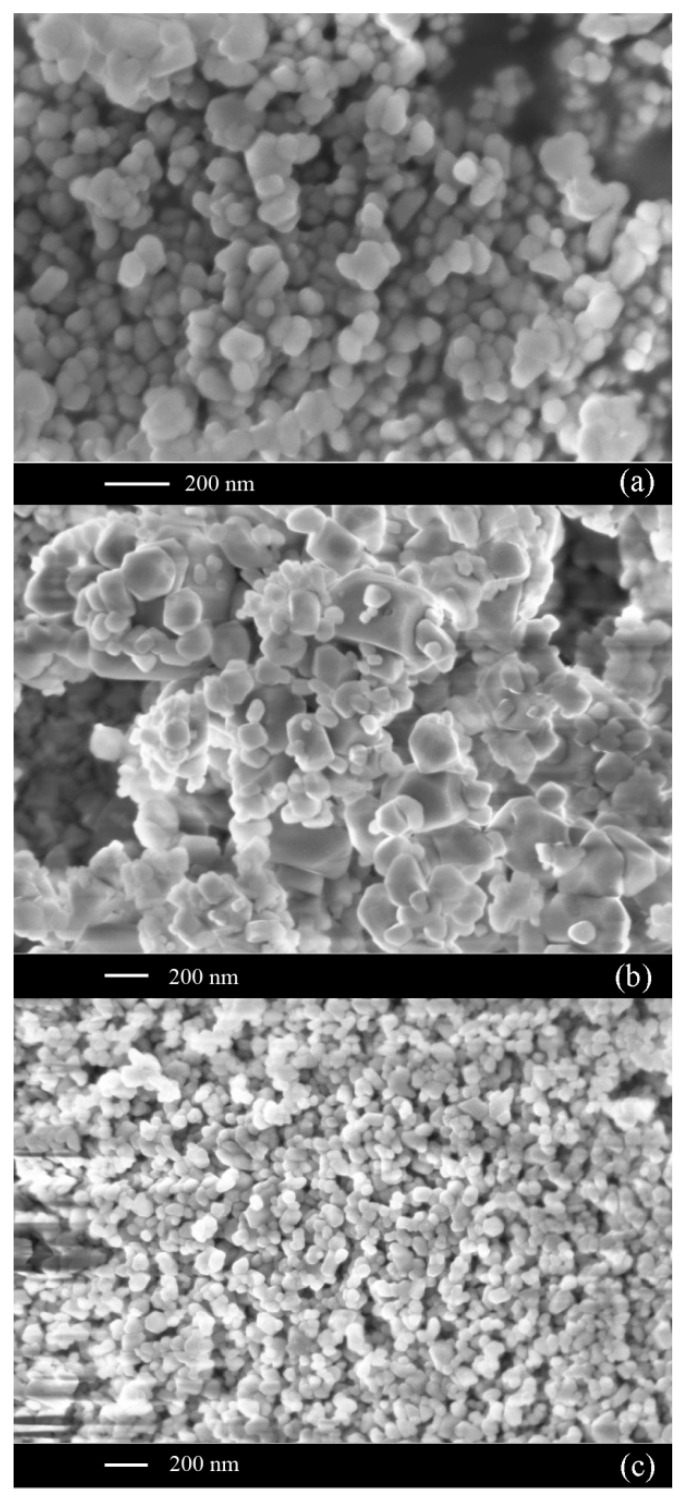
SEM photographs of CaF_2_ powders synthesized in a one-step microreactor with intensive swirled flows. Flow rates of solutions significantly influence the size of the particles—the higher the flow rate, the smaller the size: *Q*_1a_ = *Q*_1b_ = 2.1 L/min (**a**), *Q*_1a_ = *Q*_1b_ = 2.6 L/min (**b**), and *Q*_1a_ = *Q*_1b_ = 3.2 L/min (**c**).

**Figure 14:**
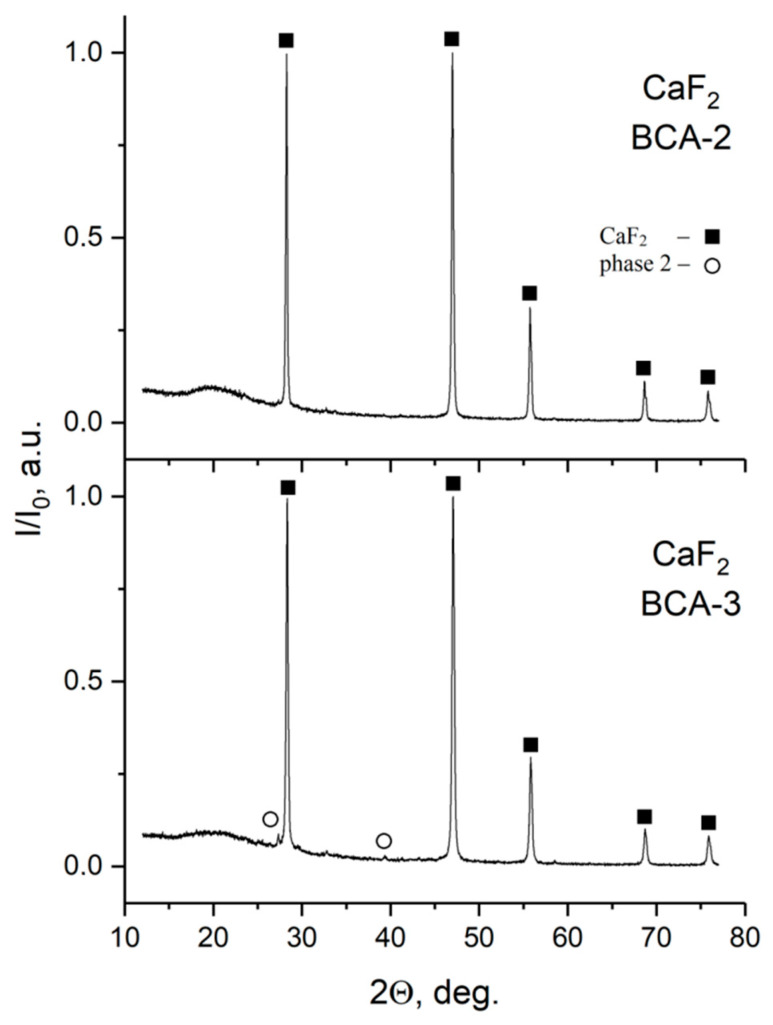
Diffractograms of products synthesized in MRISF-CC-1 *Q*_1a_ = *Q*_1b_ = 2.6 L/min (BCA-2) and *Q*_1a_ = *Q*_1b_ = 3.2 L/min (BCA-3); only CaF_2_ peaks are observed, along with the second product—KNO_3_.

Synthesis of calcium and strontium fluorides was carried out in the microreactor with a counter-current intensive swirled flow (MRISF-CC-1) [41,42]; see Figure 13 and Figure 14.

The substitution reactions carried out,

Ca(NO_3_)_2_ + 2KF ⟶ 2KNO_3_ + CaF_2_ (8)

Sr(NO_3_)_2_ + 2KF ⟶ 2KNO_3_ + SrF_2_ (9)

resulted in an immediate formation of crystalline products, CaF_2_ and SrF_2_.

Interestingly, CaF_2_ powders with an average size of 40 nm are produced at flow rates of *Q*_1a_ = *Q*_1b_ = 3.2 L/min (case BCA-3 in Figure 13). An increase in the flow rate of solutions prevents the formation of agglomerates and improves the granulometric uniformity of the powder. The absence of faceting at a low synthesis temperature (20 °C) is a sign of processes far from equilibrium.

**Figure 15:**
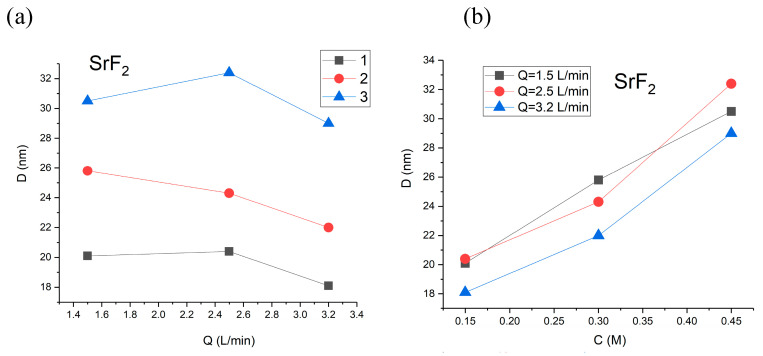
Dependence of crystalline size *D* (nm): (**a**) on the concentration of reagents (Sr(NO_3_)_2_ 0.45 M (line 1), 0.30 M (line 2), 0.15 M (line 3)) and (**b**) on the flow rates of solutions in MRISF-CC-1.

A similar effect has been obtained for SrF_2_: a decrease in crystalline size was observed for growing flow rates of solutions (Figure 15) [42]. As expected, the increase in the concentration of reagents resulted in the larger size of synthesized particles due to the higher intensity of ion collisions in the solution. The smallest particles of SrF_2_ with *D* = 18 nm have been synthesized at a Sr(NO_3_)_2_ concentration of 0.15 M and flow rates of solutions *Q*_1a_ = *Q*_1b_ = 3.2 L/min; the maximal size of particles was 32 nm at a Sr(NO_3_)_2_ concentration of 0.45 M and lower flow rates of solutions. Hence, the use of microreactors allows the control of the size of synthesized nanoparticles.

## 7. Synthesis of Nanosized Particles of Composites

**Figure 16:**
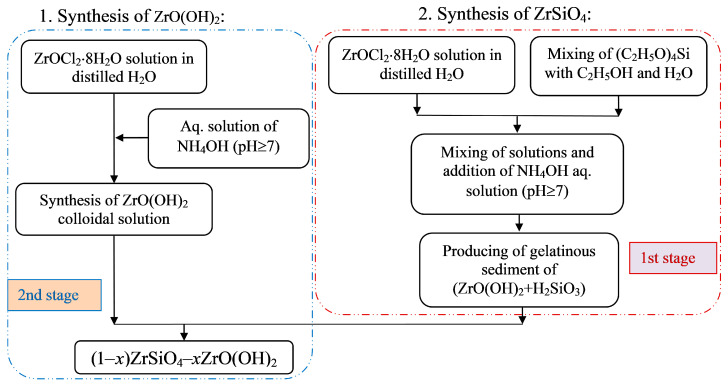
Schematic diagram of two-stage synthesis of (1 − *x*)ZrSiO_4_ − *x*ZrO(OH)_2_ composite nanoparticles in the two-stage microreactor with intensive swirled flows, MRISF-2.

**Figure 17:**
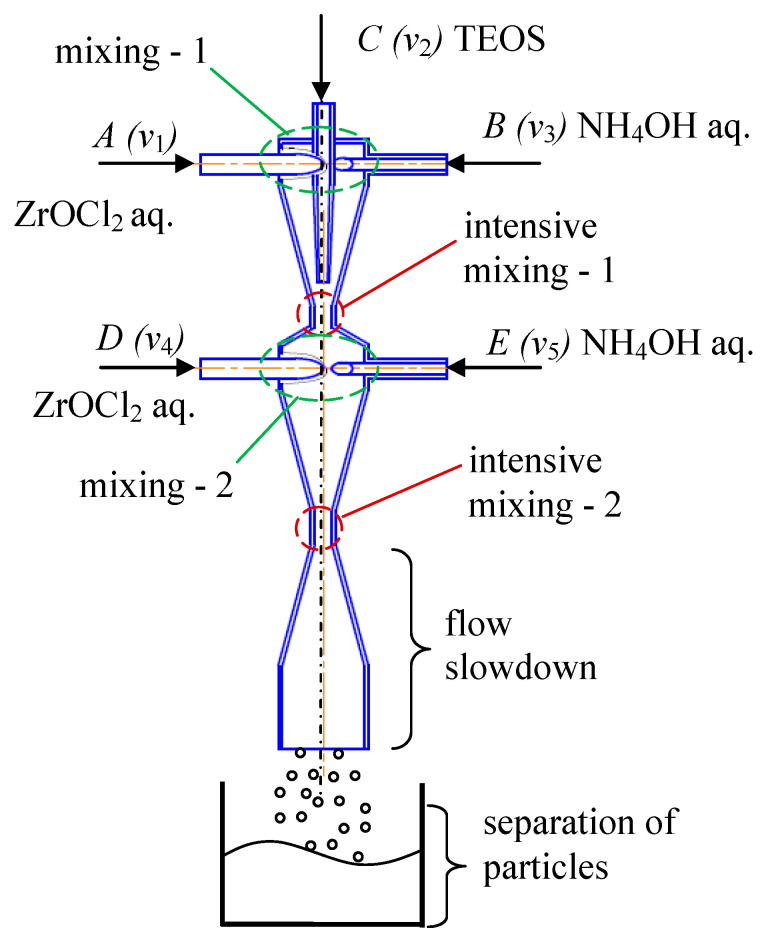
Flow feeding in the two-stage synthesis microreactor with intensive swirled flows, MRISF-2 of (1 − *x*)ZrSiO_4_ − *x*ZrO(OH)_2_. Five flows are fed simultaneously, ensuring a high throughput of the microreactor. Similarly, (1 − *x*)ZrSiO_4_ − *x*HfO_2_ composites have been produced in this microreactor.

One of the recent syntheses in a two-stage microreactor with intensive swirled flows allowed the production of nanosized particles of composite materials [46,47] (Figure 16). The first stage is shown on the right side of the diagram in Figure 16 (highlighted as a red rectangle with rounded corners), and the second stage is on the left side in Figure 16 (highlighted as a blue rectangle with rounded corners); a schematic of the feeding flows is shown in Figure 17.

The opportunities of the two-stage microreactor MRISF-2 (see Figure 6b) have been used in this case: three solutions have been fed into the first chamber of MRISF-2, and two other solutions have been fed into the second chamber; in total, five inlet pipes have been utilized simultaneously for solution feeding.

Ceramic samples (1 − *x*)ZrSiO_4_ − *x*ZrO_2_ were obtained from this precursor, as well as from (1 − *x*)ZrSiO_4_ − *x*HfO_2_, which can be used for the disposal of waste from nuclear power plants. As our studies have shown, the leaching rates of Zr, Si, radionuclides ^152^Eu, ^137^Cs, and ^90^Sr from the obtained ceramic composites (1 − *x*)ZrSiO_4_ − *x*ZrO_2_ and (1 − *x*)ZrSiO_4_ − *x*HfO_2_ were three orders of magnitude lower than from known waste disposal materials.

Currently, together with colleagues from various institutions of the Russian Academy of Sciences, several more types of materials for orthopedics (ZrO_2_ with various dopants, Y, Yb), electrically conductive ceramics ((CeO_2_)_1−*x*_(Dy_2_O_3_)*_x_*), supercapacitors (MnO_x_), membranes for solid oxide fuel cells (based on BaSrCoFe), and turbine blades for aircraft engines (NdMgAl_11_O_19_ with a magnetoplumbite structure) have been successfully synthesized in new microreactors. All synthesized materials demonstrated an exceptionally high degree of homogeneity due to the high quality of micromixing (according to the results of quantitative studies [10,12,35], ***the quality of micromixing in microreactors is 50 to 2400 times higher than in traditionally used co-precipitation stirred tank reactors***), as well as improved special characteristics (strength, hardness, electrical capacity, electrical conductivity, etc.).

## 8. Conclusions

Microscale reactors and devices for combined processes allow for the significant intensification of transfer processes (intensification of heat and mass transfer coefficients by 3–7 times in microreactors with two-phase flows and intensification of the quality of micromixing by up to 2400 times in microreactors with intensive swirling single-phase flows), which leads to a reduction in their mass and size characteristics and, most importantly, provides the ability to carry out complex processes for which a combination of high coefficients of matter and energy transfer is important.

In addition, the synthesis of inorganic functional materials in microreactors achieves several useful effects as follows: (1) a stoichiometric ratio of ions in the product, ensuring the absence of impurities; (2) synthesis of nanoparticles with reduced sizes due to the short residence time in the apparatus; (3) high productivity and continuity of the process allow for an easy transition to the industrial level without scaling (due to the use of parallel apparatuses); (4) high conversion values are ensured, for example, during the synthesis of neodymium magnesium hexaaluminate (NdMgAl_11_O_19_, the work was carried out with colleagues from the N.S. Kurnakov Institute of General and Inorganic Chemistry), the conversion for Nd was 99.999%, and for Al, it was 99.891% (results of our own studies, which is in preparation for publication). The completeness of the reaction is critical, both in terms of a significant reduction in the load on treatment facilities and in terms of increasing the product yield.

These beneficial effects have been found to be achievable in microreactors due to the following controllable factors: (1) the flow rate of each reagent solution; (2) the level of specific energy dissipation in the reaction zone; (3) the quality of micromixing, mixing time, and residence time; (4) the pH and *T* values in the reaction zone (if necessary, these parameters can be varied in different zones of the apparatus).

To these advantages, a very low level of energy consumption should be added: no more than 10–15 W is spent on each flow of solution in the microreactor.

An important feature of the developed microreactors for industrial application is the successful combination of several factors (along with high micromixing quality indicators and the possibility of synthesizing a wide class of functional materials) such as: (1) a high productivity of 420 L/h ≈ 10 m^3^/day (for suspension), which at a particle concentration of 20–30 g/L, corresponds to productivity for the solid phase—particles in the suspension—of approximately 200–300 kg/day (6000–9000 kg/month); (2) compactness of the unit, where one device with piping takes up an area of no more than 0.3 m^2^; up to five units can easily be placed on the area of a writing table (desk); (3) the power consumption of one device (for two flows) is no more than 30 W.

Hence, even the single microreactor of an MRISF type is suitable for large-scale production (up to 9000 kg/month of nanoparticles), and a set of five to six such microreactors is able to produce up to 45–54 tons/month.

It is known that intensive (rapid) micromixing provides increased stability of nanoparticles because the final suspension contains predominantly anti-solvent, which depresses the rate of Ostwald ripening [28].

The above advantages allow one to be quite optimistic about the prospects for transferring microreactor synthesis to an industrial scale, and industrial applicability was mentioned in a recent review paper on the use of microreactors of the central-collision type [48], which should inspire companies to consider microreactor technology for massive production. A variety of synthetic results for functional nanoparticles, including nickel, platinum–cobalt alloys, gold and silver nanoshells, patchy particles, core–shell clusters, and metal–organic frameworks produced in the microreactor of the central-collision, are presented in [48]; fourteen streams (seven for each solution) move radially towards the center of the microreactor and collide into a single stream at the center. It was particularly shown that some features are only feasible in microreactors, among them, trapping reaction intermediates in a sequential reaction process and rapidly changing the reaction temperature [48].

Of course, the remaining stages of the synthesis process also require the attention of researchers. We are currently developing equipment for continuous hydrothermal treatment (a laboratory setup with a capacity of up to 200 mL/min and a temperature of up to 152 °C has been manufactured and is being adjusted) [49].

Some novel papers devoted to the analysis of the micromixing quality in microreactors have been published recently (not included in this review) and could be interesting for readers, such as microreactors with complex geometry [50], an acoustic-assisted microreactor [51], and an AI-assisted experimental study of a micro-coiled flow inverter [52].

It should be noted that MRFIJ has limitations similar to those mentioned above for impinging-jets mixing devices in general: too high a velocity of jets results in excess kinetic energy of jets, and this, in turn, leads to the precocious rupture of the liquid sheet. Thus, the performance of the impinging-jets reactors (including MRFIJ) is limited by the diameter of jets (usually not larger than 2.0–2.5 mm) and by the velocity of jets (<5–8 m/s).

Microreactors with intensively swirling flows also have some limitations, such as (i) their suitability for very fast reactions (with a reaction time < 10 ms); (ii) it is better to preheat solutions of reagent to the reaction temperature; (iii) the ratio of flow rates supplied through various inlet pipes is limited approximately by three to five inlet pipes, but this issue could be circumvented by a distributed liquid supply through two to six inlet pipes.

MRFIJ-type and MRISF-type microreactors cannot be used for the synthesis of organic products due to short residence times. Moreover, commonly, some thermal treatment (either in a dry state, such as annealing, or hydrothermal treatment) significantly influences the structure and morphology of particles; however, if the amorphous precursor produced in MRISF-type microreactors has a proper stoichiometric ratio of elements, a risk of formation of impurities is avoided.

For the microreactor synthesis, it is imperative to conduct a comprehensive review of the potential for clogging, especially in microchannels smaller than 0.1–0.2 mm, alongside evaluating the residence time in the active micromixing zone, ensuring it is somewhat greater than the reaction duration.

On the other hand, some synthesis processes need quite narrow pH values, and these should be defined and maintained in the microreactor in order to obtain the necessary product.

Moreover, it is strongly recommended to handle the suspension with nanosized particles immediately after synthesis in order to avoid rapid agglomeration and aggregation.

These recommendations are aimed at obtaining nanoparticles with the necessary properties.

The scale-up challenges for microreactors can be attributed to fouling and/or clogging. For some types of microreactors, like the T-mixers, Y-mixers, Hartridge–Roughton reactor, and two-phase segmented flow microreactors, this problem can arise in the case of small channels (about 0.1…0.2 mm). The microreactors with free impinging jets (MRFIJ) and microreactors with intensive swirled flows (MRISF) discussed in this paper do not have such limitations due to the relatively large diameter of the jets (for MRFIJ, the diameter of the jets is 0.5…2.0 mm), or relatively large diameter of the neck (*d* = 2.0…2.4 mm for MRISFs).

Intensive mixing prevents uniformity challenges in intensive regimes. The costs of microreactors are many hundreds of times smaller compared to the usual scale reactors. Energy consumption in absolute values is very small (not more than 30 W for two inlet flows), and the produced amount (200–300 kg/day) is comparable with that of a 1 m^3^ stirred tank reactor (about 1000–3000 W). Consequently, an initial assessment reveals cost advantages associated with the microreactors detailed in this review, in addition to superior quality of resultant nanoparticles.

Future research should be aimed, in our opinion, at the creation of effective devices for the continuous separation of nanoparticles from mother liquor, s well as continuous drying, hydrothermal synthesis, and thermal treating.

## Figures and Tables

**Table 2:** Some examples of inorganic materials synthesized in microreactors with intensive micromixing.

Name of the Substance Material	Specific Properties, Areas of Application	References
BiFeO_3_	Multiferroic and photovoltaic properties	[39]
GdFeO_3_	Contrast materials for magnetic resonance tomography	[8,9]
Fe_3_O_4_	Electronic “ink”, heat sources in biomedicine, and fertilizers for agriculture	[40]
TiO_2_ (+Nd_2_O_3_)	Materials with photocatalytic activity	[40]
CaF_2_	Luminescent and optical materials	[41]
SrF_2_	Materials for photonics	[42]
Y_3_Al_5_O_12_ (YAG)	Solid-state lasers	[40]
CoFe_2_O_4_	Magnetic materials, for the adsorption of Al^3+^, Zn^2+^, catalysts, electrodes of lithium-ion power sources, and electrodes of fuel cells	[40]
ZrO_2_ + Y_2_O_3_	Materials for endo- and orthoprosthetics	[43]
hydrosilicate nanoscrolls of composition Mg_3_Si_2_O_5_(OH)_4_	Nanocomposite components with a wide range of applications	[40]
Bi_2_O_3_–Fe_2_O_3_–WO_3_ (pyrochlore structure)	(1) Photovoltaic cells; (2) photocatalysts; (3) coatings for anodes in photoelectrochemical cells for generating hydrogen in the process of water decomposition; (4) materials for spintronics; (5) matrices for immobilization of radioactive waste; (6) solid electrolytes in solid oxide fuel cells	[44]
BiVO_4_	Materials with photocatalytic activity	[45]
ceramic composites based on (zircon) (1 − *x*)ZrSiO_4_ − *x*ZrO_2_	Composite materials for the disposal of nuclear power plant waste	[46,47]

## Data Availability

No new data were created or analyzed in this study. Data sharing is not applicable to this article.

## Nomenclature

[A]: [B]	concentrations of reagents A and B, mol/m^3^
*D*	diffusion coefficient, m^2^/s
*D*_A_, *D*_B_	diffusion coefficients of molecules or particles of reagents A and B, m^2^/s
*k*_eff_	effective rate constant, m^3^/(mol s)
*k*_react_	true rate constant of chemical reaction, m^3^/(mol s)
*k*_D_	rate constant of diffusion transfer, m^3^/(mol s)
*L*	characteristic size of apparatus, m
Pe	Peclet number, Pe = *UL/D*
*r*_A_, *r*_B_	radii of particles A and B, m
Sc = ν/*D*	Schmidt number
*t_m_*	micromixing time, s
*U*	characteristic velocity of liquid movement, m/s
*w*	rate of chemical reaction, mol/(m^3^ s)
ε	specific energy dissipation rate, W/kg
ν	kinematic viscosity, m^2^/s
